# Phosphatidylserine and phosphatidylethanolamine regulate the structure and function of FVIIa and its interaction with soluble tissue factor

**DOI:** 10.1042/BSR20204077

**Published:** 2021-02-03

**Authors:** Tanusree Sengupta, Tilen Koklic, Barry R. Lentz, Rinku Majumder

**Affiliations:** 1 Department of Chemistry, Sri Sivasubramaniya Nadar College of Engineering, Kalavakkam, India; 2 Laboratory of Biophysics (EPR Center), Jozef Stefan Institute, Jamova 39, 1000 Ljubljana, Slovenia; 3 Department of Biochemistry and Biophysics, CB#7260, University of North Carolina at Chapel Hill, Chapel Hill, NC 27599-7260, U.S.A.; 4 Department of Biochemistry and Molecular Biology, Louisiana State University Medical School, 7144 MEB, New Orleans, LA 70112

**Keywords:** Blood coagulation, Factor VIIa, Soluble tissue factor, Factor X, Phosphatidylserine, Phosphatidylethanolamine

## Abstract

Cell membranes have important functions in many steps of the blood coagulation cascade, including the activation of factor X (FX) by the factor VIIa (FVIIa)-tissue factor (TF) complex (*extrinsic X_ase_*). FVIIa shares structural similarity with factor IXa (FIXa) and FXa. FIXa and FXa are regulated by binding to phosphatidylserine (PS)-containing membranes via their γ-carboxyglutamic acid-rich domain (Gla) and epidermal growth-factor (EGF) domains. Although FVIIa also has a Gla-rich region, its affinity for PS-containing membranes is much lower compared with that of FIXa and FXa. Research suggests that a more common endothelial cell lipid, phosphatidylethanolamine (PE), might augment the contribution of PS in FVIIa membrane-binding and proteolytic activity. We used soluble forms of PS and PE (1,2-dicaproyl-sn-glycero-3-phospho-l-serine (C6PS), 1,2-dicaproyl-sn-glycero-3-phospho-ethanolamine (C6PE)) to test the hypothesis that the two lipids bind to FVIIa jointly to promote FVIIa membrane binding and proteolytic activity. By equilibrium dialysis and tryptophan fluorescence, we found two sites on FVIIa that bound equally to C6PE and C6PS with *K*_d_ of ∼ 150–160 μM, however, deletion of Gla domain reduced the binding affinity. Binding of lipids occurred with greater affinity (*K*_d_∼70–80 μM) when monitored by FVIIa proteolytic activity. Global fitting of all datasets indicated independent binding of two molecules of each lipid. The proteolytic activity of FVIIa increased by ∼50–100-fold in the presence of soluble TF (sTF) plus C6PS/C6PE. However, the proteolytic activity of Gla-deleted FVIIa in the presence of sTF was reduced drastically, suggesting the importance of Gla domain to maintain full proteolytic activity.

## Introduction

An essential feature of blood coagulation is the formation of complexes between serine protease coagulation factors and their corresponding membrane-bound cofactors. Tissue factor (TF) is a 47-kDa protein expressed on the surface of several types of cells that do not contact blood [1]. Tissue damage exposes TF to blood; exposed TF binds to factor VIIa (FVIIa) to form the TF–FVIIa complex in which membrane-bound TF is the FVIIa cofactor [2]. The activated platelet surface provides the membrane lipids that are involved in amplification and propagation phases of coagulation, but damaged endothelial cell membranes are the primary membranes available during the initiation phase [3,4]. Both types of membranes expose the lipid phosphatidylserine (PS), but the latter membrane type is the site of action of the TF–FVIIa complex [3]. Although FX activation by TF–FVIIa is complicated by FVIIa auto-activation [5,6], the rate of FX activation by FVIIa–TF on the endothelial cell membrane surface is enhanced by ∼10^4^–10^5^-fold relative to activation by FVIIa alone [5]. Thus, the endothelial cell membrane has a key function in the complicated process of initiation of blood coagulation.

The participation of membranes and lipids in TF–FVIIa activation of FX (or FIX) has long been recognized. However, unlike the function of membranes and lipids in FXa-FVa activation of prothrombin [7] and in FIXa-FVIIIa activation of FX [8], the actions of membranes and lipids are enigmatic regarding TF–FVIIa activity. FVIIa is structurally similar to FXa and FIXa [9] and, like FXa and FIXa, FVIIa binding to membranes requires an N-terminal γ-carboxyglutamic acid-rich domain (Gla domain). However, FXa and FIXa both have reversibly bound extrinsic protein cofactors (FVa and FVIIIa), whereas TF is a Type I intrinsic protein cofactor permanently anchored to the endothelial cell membrane by a *trans*-membrane domain [2]. This unique feature of TF has impeded characterization of the activities of specific lipids in regulating the FVIIa (FVII)–TF complex.

The serine protease and cofactor activities of the FXa–FVa and FIXa–FVIIIa complexes are regulated mainly by the platelet PS exposed on activated platelets [8,10] and, to a lesser extent, by phosphatidylethanolamine (PE) [7]. Short chain *soluble* (i.e., having a high critical micelle concentration (CMC)) phospholipids have enabled dissection of membrane interactions with FXa and FVa. Dicaproyl-PS (1,2-dicaproyl-sn-glycero-3-phospho-l-serine, C6PS), a stand-in for PS, binds to specific sites on FXa to increase its activity [7] and to a PS-specific site in the C1 domain of FVa to enhance its affinity for FXa [11]; thus, C6PS enables assembly of an active FXa–FVa complex in solution [10]. C6PS and 1,2-dicaproyl-sn-glycero-3-phospho-ethanolamine (C6PE) (a PE substitute) do not occur physiologically, but they are powerful tools for locating and characterizing the specificities and the physiological functions of lipid-regulatory sites in FXa, FIXa, and their cofactors FVa and FVIIIa [7,11–13].

In contrast, TF must be studied in native or reconstituted membranes. Although investigators have recognized the important function of PS in FXa activation by FVIIa–TF, there are contradictory reports about the mechanism of the effects of PS on activation of FX by FVIIa–TF. One view is that the membrane has no activity in delivering the substrate (FX) to the complex [13,14]. Another view is that interaction of FX with a PS-containing membrane delivers FX to the complex in an appropriate conformation to maximize *k*_cat,app_ [14]. This second view is supported by observations that FXa undergoes significant conformational changes on binding to membranes [8,15] or on binding to soluble PS in solution [16]. This view also appears to be consistent with a crystal structure of FVIIa bound to a soluble recombinant form of TF (residues 1–219; rsTF) [17] that was cleaved by subtilisin to promote crystallization [18].

Besides the surface delivery of FX or removal of FXa, the endothelial membrane might also contribute to FVIIa activity by properly aligning FX with the FVIIa active site or by regulating FVIIa–TF complex formation. These mechanisms contribute to FXa–FVa activity on platelet-derived membranes [10,11,19,20]. Thus, to decipher the exact function(s) of phospholipid in regulating FVIIa activity, we used soluble short-chain phospholipids to address the following questions: (1) Do C6PS or C6PE increase the activity of FVIIa in solution? (2) Do C6PS or C6PE increase affinity of soluble TF (rsTF) for FVIIa in solution? (3) Is binding of C6PS or C6PE to rsTF required for activity of the FVIIa–rsTF complex in solution? Our results are summarized as follows:

1. Short-chain soluble PS and PE bind to FVIIa with an apparent dissociation constant of 150 ± 100 μM, as measured by FVIIa structural changes, proteolytic activity, and amidolytic activity. Fitting several datasets, we identified two lipid binding sites on FVIIa, that bind either soluble PS or PE with similar dissociation constants.
2. Saturating proteolytic activity of FVIIa was approximately 5-times greater in the presence of soluble PS compared with soluble PE, and approximately 15-times greater when both lipids were present. Thus, the two lipids appeared to act synergistically. However, the proteolytic activity of FVIIa was highest, by approximately 100 times, in the presence of rsTF and both lipids.
3. Soluble TF binds to FVIIa and Gla domainless FVIIa (des Gla-FVIIa) with similar dissociation constants of approximately 2 nM at saturating concentrations of soluble lipids. However, the saturating proteolytic activity of FVIIa was approximately 200-times higher than the activity of the des Gla-FVIIa.

In conclusion, soluble lipids (PS and PE) facilitate tight binding (*K*_d_ = 2 nM) of both FVIIa and des Gla-FVIIa to rsTF; however, the Gla domain of FVIIa is essential for the FVIIa–rsTF complex to achieve physiologically relevant activity.

## Materials and methods

### Materials

Human FVIIa and des Gla-FVIIa were purchased from Enzyme Research Laboratories (South Bend, IN). rsTF was a generous gift from Professor James Morrissey (University of Michigan). The sodium salts of C6PS and C6PE were purchased from Avanti Polar Lipids (Alabaster, AL). The FXa-specific substrate N-2-benzyloxycarbonyl-d-arginyl-l-arginine *p*-nitroanilide dihydrochloride (S-2765) was purchased from Diapharma (West Chester, OH). All other chemicals were ACS reagent grade or the best available grade; all solvents were HPLC grade.

### Methods

#### Fluorescence measurements

Intrinsic tryptophan fluorescence of FVIIa as a function of increasing concentration of soluble lipid was measured as described in [20] using a SPEX® FluoroLog-3 spectrofluorometer (Horiba Jobin Yvon Inc., Edison, NJ) with excitation wavelength at 285 nm (band-pass 4 nm) and emission recorded at 340 nm (band-pass 4 nm).

#### Proteolytic activity assay

Proteolytic activity of FVIIa was followed by measuring the rate of generation of FXa from FX according to the method described in [12] with slight modification. Briefly, 100 nM FVIIa was incubated with different concentrations of C6PS in appropriate volume of buffer (20 mM Tris with 150 mM NaCl, 5 mM CaCl_2_ and 0.6% PEG, pH 7.5) in the wells of a microtiter plate at 37°C for 5 min. A total of 300 nM FX and 1 mM of FX specific synthetic substrate S-2765 was added to it and absorbance at 405 nm was recorded for 5 min using a VersaMax tunable micro plate reader (Molecular Devices) at 37°C. The amount of FXa produced was determined from the initial rate of substrate cleavage using a standard curve prepared with active-site-titrated FXa as described earlier [21].

#### Amidolytic activity assay

The amidolytic activity (absorbance *versus* time) of FVIIa in the presence of various concentrations of C6PS or C6PE was determined using a synthetic chromogenic substrate, Leu-PHG-Arg-pNA.AcOH (Aniara, West Chester, OH) according to the method described earlier with slight modifications [22]. Amidolytic activity was measured in buffer (20 mM Tris with 150 mM NaCl, 5 mM CaCl_2_ and 0.6% PEG, pH 7.5) containing human FVIIa (100 nM), 1 mM chromogenic substrate, and varying concentrations of C6PS. The time course of absorbance yielded the amidolytic activity of FVIIa. A small volume of the assay buffer was first incubated in a 96-well microplate at 37°C. Human FVIIa was next allowed to bind C6PS or PE by adding both to the buffer in the well and equilibrated for 5 min at 37°C. Finally, the substrate was added to that at a final concentration of 1 mM. The absorbance at 405 nm was recorded every 20 s for 5 min using a VersaMax tunable microplate reader (Molecular Devices) to obtain the initial rate of substrate hydrolysis. Triplicate determinations were obtained for each lipid concentration in each of two independent experiments. Activity in the presence of lipids was recorded as a percent of activity in the absence of lipid.

#### Analysis of C6PX binding

Binding of lipid to FVIIa was assessed indirectly by recording the appropriate observable (proteolytic activity, amidolytic activity, intrinsic fluorescence) as a function of soluble lipid (C6PS or C6PE) concentration over a broad range below the CMC of the soluble lipid. The CMC was independently assessed for each conditions following pyrene fluorescence as a function of increasing lipid concentration [23]. Simple binding models were fitted to these experimental data to estimate the apparent dissociation constants for binding of FVIIa to C6PS or C6PE, as described in the Appendix, using the appropriate assumption that [C6PX]_free_ ≈ [C6PX] [7]. Analysis of binding curves is described in detail in the Appendix.

#### Binding of C6PS or C6PE to FVIIa by equilibrium dialysis

The stoichiometry of soluble C6PS and C6PE binding to FVIIa was estimated by equilibrium dialysis measurements. Experiments were performed using 2.0 ml Teflon dialysis cells (Harvard Apparatus, Holliston, MA) with the two cells separated by a 2000 molecular weight cut-off membrane. The total lipid concentration, [L]_tot_, and total protein concentration, [P]_tot_, must be large enough to allow accurate determination of differences in organic phosphorous between the two chambers but [L]_tot_ must be below the lipid CMC.

#### Computational procedure used for global fitting of all datasets

The details of the computational procedures are described in the ‘Results’ section.

## Results

### Effect of soluble lipids on structural response of FVIIa and des Gla-FVIIa measured by intrinsic tryptophan fluorescence

**Figure 1:**
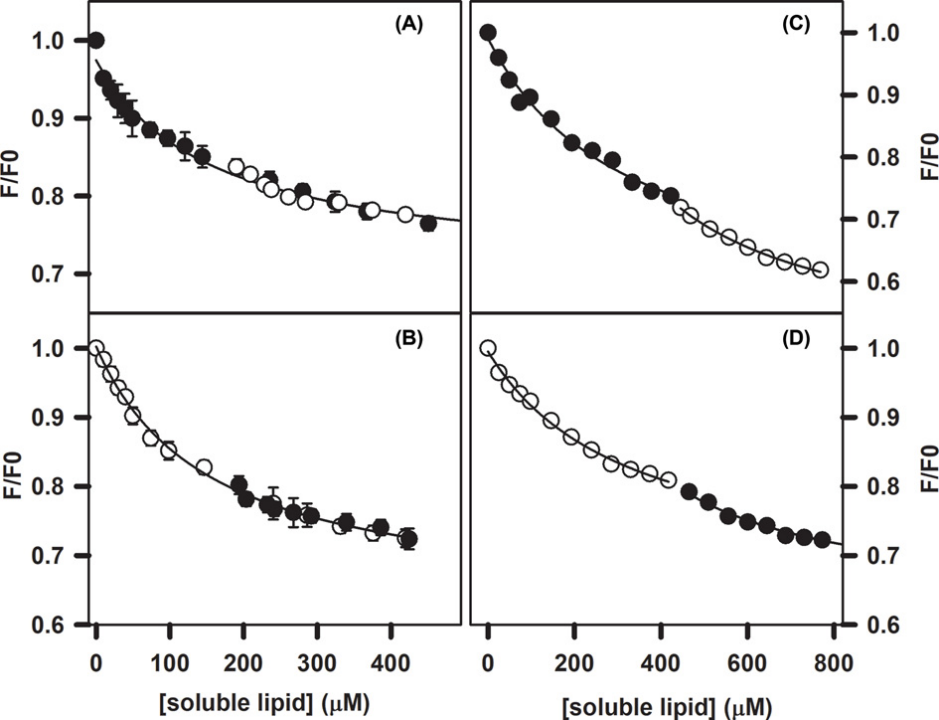
C6PS and C6PE bind with similar affinity to FVIIa Effect of C6PS and C6PE on intrinsic tryptophan fluorescence intensity of 100 nM FVIIa (**A,B**) and des Gla-FVIIa (**C,D**) in 50 mM Tris, 175 mM NaCl, 5 mM CaCl_2_, 0.6% PEG, pH 7.5 and 23°C. Relative fluorescence intensity (F/F_0_) is plotted as a function of added C6PS (•) or C6PE (○). (A) Titration of FVIIa by C6PS from 0 to 558 μM yielded an estimated *K*_d_ = 160 ± 30 μM. FVIIa pre-incubated with 200 µM C6PS was also titrated with C6PE, as shown in open circles (○). The combined data (C6PS and C6PE) were best described with *K*_d_ = 171 ± 30 μM. (B) Titration of FVIIa by C6PE from 0 to 420 μM (open circles) *K*_d_ = 154 ± 14 μM. C6PS was also added to FVIIa pre-incubated with 200 µM C6PE (closed circles). The combined data (C6PS and C6PE) were best described with *K*_d_ = 151 ± 12 μM. (C) C6PS (•) was added to 100 nM des Gla-FVIIa up to a concentration of 417 μM (apparent *K*_d_ = 319 ± 83 μM) after which C6PE (○) was added up to a total lipid concentration of 770 μM (apparent *K*_d_ = 354± 63 μM); (D) the intrinsic tryptophan fluorescence plotted against increasing amount of C6PE (○) and then against increasing amount of C6PS (•). In all cases, apparent *K*_d_’s were estimated using a single-site binding model with *n* =1, ΔF_sat_ as a floating parameter, and [C6PS/E]_free_ taken as [C6PS/E]_tot_. These values are collected in Tables A1 and A2 in the Appendix.

The intrinsic tryptophan fluorescence intensity of FVIIa decreased with the addition of increasing amounts of C6PS and C6PE (Figure 1). The binding curves in both cases were reasonably well described by a single-site binding model (see Appendix), from which estimated apparent dissociation constants (*K*_d_’s) for binding of C6PS and C6PE were found to be 160 ± 30 and 154 ± 14 µM, respectively (Appendix, Table A1). Clearly, the intrinsic fluorescence of FVIIa responds equivalently to both lipids. This equal response could mean either (1) that a common site recognizes the two lipids interchangeably (same *K*_d_’s and responses), or (2) that multiple distinct sites recognize these two lipids with equivalent *K*_d_’s and responses. To distinguish between these possibilities, we partially titrated FVIIa with C6PS and then further titrated with C6PE (Frame A of Figure 1) or performed the reverse experiment by titrating with C6PS after titrating with C6PE (Frame B of Figure 1). Fitting and analyses of the data from these two mixed-lipid experiments revealed comparable apparent *K*_d_’s and changes in intrinsic tryptophan fluorences ΔF_sat_ for both single-lipid and two-lipid titrations for both lipids. These results support the interpretation that the fluorescence-altering site or sites is/are equally well occupied by C6PS and C6PE, with similar ensuing responses, but they do not establish the number of sites.

We also tested other soluble lipids, such as 1,2-dicaproyl-sn-glycero-3-phosphocholine (C6PC) and C6DPS, and observed very week binding (data not shown). These other lipids were also found to have no effect on the proteolytic activity of full-length FVIIa, indicating that the influence of soluble lipids on FVIIa activity is specific.

### Effect of the FVIIa–Gla domain on lipid binding

As for intact FVIIa, two type of experiments were also performed with a des Gla–FVIIa (Figure 1). C6PS and C6PE bind to des Gla–FVIIa with *roughly* the same apparent *K*_d_’s (319 ± 83 µM for C6PS and 326 ± 47 µM for C6PE) (Frames C and D of Figure 1 and Table A2 of Appendix). Absolute fluorescence of des Gla-FVIIa were not much different from the full-length FVIIa, however, the mixed lipid titration presented somewhat different result. Unlike for the intact protein, elimination of the Gla domain resulted in a slight discontinuity in the fluorescence response when the concentration of one lipid was fixed but titration continued with the other lipid. The single site model introduced for describing the data in Figure 1 (Eq. 3 in the Appendix) could not describe the discontinuity in the datasets for mixed lipid titrations with des Gla–FVIIa. The failure of this model rules out the possibility that two-independent sites on des Gla–FVIIa responds equally to PS and to PE, as we found for intact FVIIa. The single-site model described well the initial portions of each titration in which only C6PS (Frame C, Figure 1) or C6PE (Frame D, Figure 1) was added to des Gla–FVIIa. The parameters defined by these single-site fits to the initial single-lipid titrations are summarized in the first and last rows in Table A2 in the Appendix.

### Response of FVIIa proteolytic activity to soluble lipids

**Figure 2:**
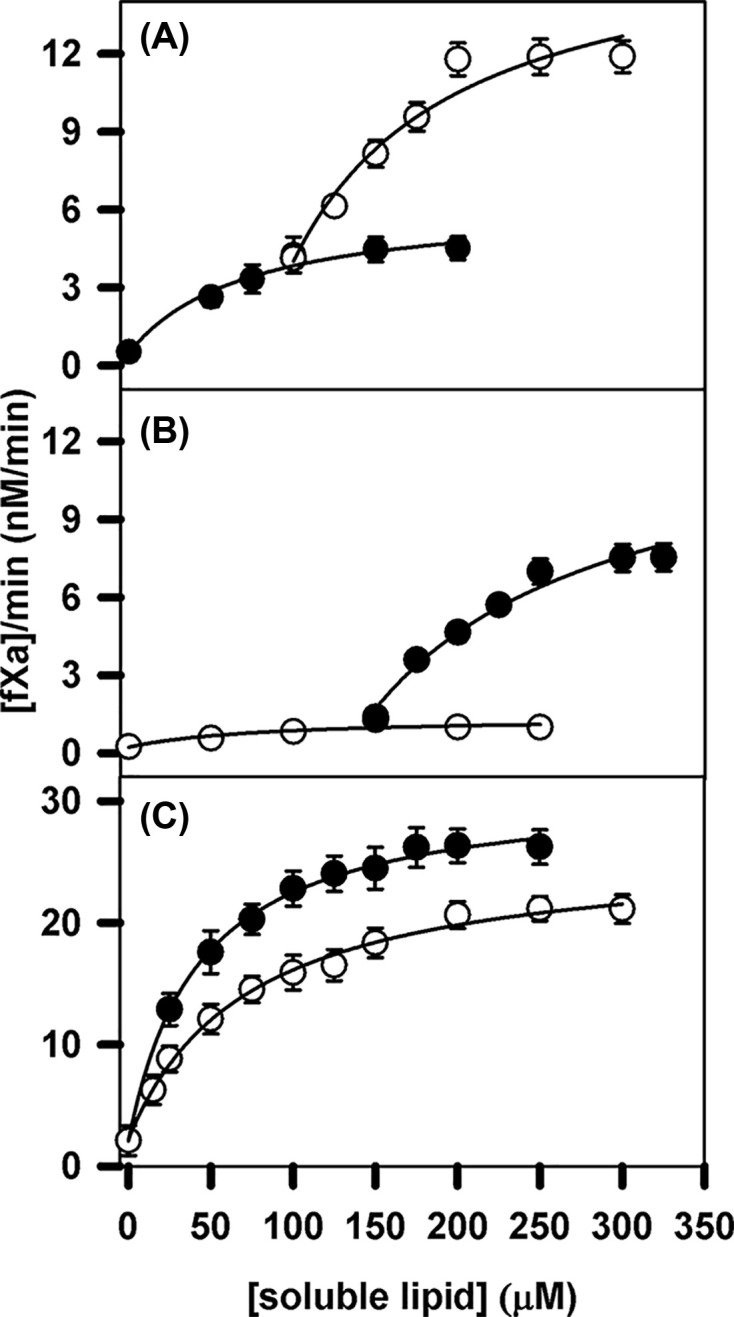
Effect of C6PS and C6PE on the proteolytic activity of FVIIa in the presence and absence of sTF In frames (**A,B**), the rate of 300 nM FX to FXa conversion by 100 nM FVIIa in an appropriate buffer (20 mM Tris with 150 mM NaCl, 5 mM CaCl_2_ and 0.6% PEG, pH 7.5) is reported as detected with 1 mM of FXa synthetic substrate S-2765 at 37°C. The lines through the data were obtained using a simple single-site model. (A) FVIIa proteolytic activity in response to binding of C6PS in the absence of C6PE (•) or of C6PE in the presence of 100 μM C6PS (○). (B) FVIIa proteolytic activity in response to binding of C6PE in the absence of C6PS (○) or of C6PS in the presence of 100 μM C6PE(•). (C) Increase in FVIIa (2.5 nM) proteolytic activity against 150 nM FX in the presence of 10 nM rsTF as a result of titration with C6PS (•) and (B) C6PE (○).

Having demonstrated sensitivity of FVIIa and des Gla-FVIIa structure towards soluble lipid binding with apparent *K*_d_’s of approximately 150–300 µM, (as reflected in intrinsic fluorescence), we next monitored conversion of FX (300 nM) to FXa by FVIIa (100 nM) in the presence of increasing concentrations of C6PS and C6PE (Figure 2A,B). Proteolytic activity increased with titration by C6PS or C6PE alone with apparent *K*_d_’s of 72 ± 30 µM for C6PS and 83 ± 106 μM for C6PE (Table A3). When we performed this experiment with initial addition of 150 µM C6PE followed by titration with C6PS, the apparent *K*_d_ was essentially the same (84 ± 22 µM) with a much larger change in saturating activity (9.5 ± 1 nM/min). Titration with C6PE in Frame A at a fixed concentration of C6PS (100 µM) yielded an apparent *K_d_* of 83 ± 40 µM with a change in saturating activity of 12 ± 2 nM/min) (Table A3). Clearly, C6PS was a tad more effective than C6PE in promoting proteolytic activity of FVIIa, but the highest proteolytic activity of FVIIa was achieved in presence of both lipids simultaneously. This indicates the cooperative influence of C6PS and C6PE in promoting the proteolytic activity of FVIIa.

Next, we considered the influence of rsTF on the response of FVIIa proteolytic activity to C6PS and C6PE. These data are shown in Figure 2C with results recorded in Table A3. Once again, the solid lines through the data represent a single-site binding model from which we estimated apparent *K*_d_ = 43 ± 3 µM, with saturating activity *a* = 29.1 ± 0.6 nM/min for PS; and *K*_d_ = 72 ± 8 µM, with saturating activity *a* = 23.9 ± 0.7 nM/min for PE. Thus, it is clear that rsTF enhanced the activation by ∼100-fold of FVIIa by both lipids and eliminated the somewhat larger influence of C6PS. As for FXa and FIXa [7,10–12,13,20], the FVIIa cofactor is also regulated by lipids. Note that saturating activity of approximately 30 nM/min was obtained in presence of 10 nM rsTF, and 2.5 nM FVIIa (40-times lower than in frames A and B), and 150 nM FX (2-times lower than in frames A and B).

### Effect of rsTF on FVIIa and des Gla-FVIIa proteolytic activity in presence of soluble lipids

**Figure 3:**
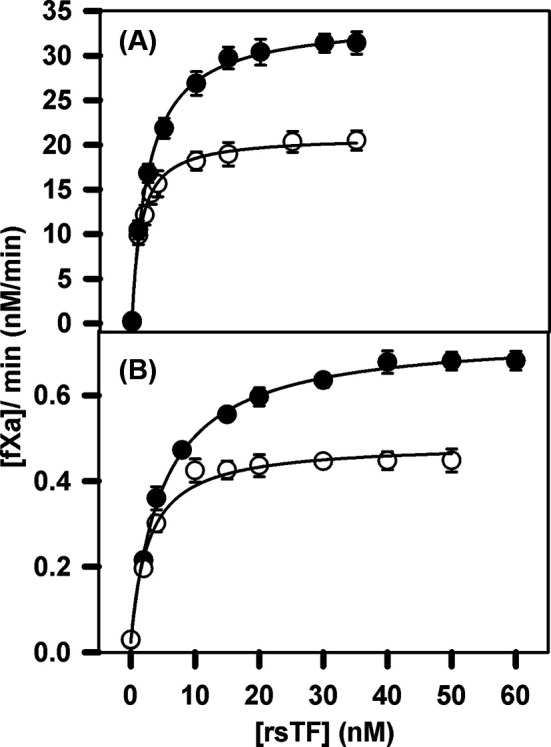
Interaction of rsTF with FVIIa and des Gla-FVIIa in presence of soluble lipids (A) Rate of proteolytic activation of 150 nM FX by 2.5 nM FVIIa in the presence of 100 µM C6PS (•) or C6PE (○) was titrated against increasing concentration of rsTF in an appropriate buffer (20 mM Tris with 150 mM NaCl, 5 mM CaCl_2_ and 0.6% PEG, pH 7.5) at 37°C following the method described in the ‘Materials and methods’ section. Activation was monitored using 1 mM FXa substrate S-2765. The apparent *K*_d_’s of the interaction of rsTF with FVIIa were 2.6 ± 0.6 nM with C6PS and 1.3 ± 0.5 nM with C6PE. (B) Rate of proteolytic activation of 300 nM FX by 10 nM des Gla-FVIIa in the presence of 100 µM C6PS (•) or C6PE (○) was titrated against increasing concentration of rsTF. The apparent *K*_d_’s of the interaction of rsTF with des Gla–FVIIa were 5.0 ± 0.3 nM with C6PS and 2.7 ± 0.2 nM with C6PE.

FVIIa interacts with membrane-located TF much more tightly as PS content increases [24]. The result in Figure 2C suggests a lipid-dependent interaction between FVIIa and rsTF even in solution. To estimate the strength of this interaction, we titrated the effect of rsTF on FVIIa (2.5 nM) activation of 150 nM FX in the presence of 100 μM C6PS or C6PE, with the results recorded in Figure 3A. Addition of rsTF to FVIIa enhanced the ability of both C6PS (•) and C6PE (○) to promote FVIIa proteolytic activity in activation of FX. Soluble lipids alone promoted FVIIa proteolytic activity (Figure 2, Frames A and B), reaching saturating activity of approximately 5, and 1 nM/min in presence of saturating amount of C6PS and C6PE, respectively. Addition of rsTF enhanced the lipid mediated proteolytic activities to approximately 30 and 20 nM/min, respectively; however, at 40-times lower amount of FVIIa and 2-times lower amount of FX, thus increasing saturating activity more than 100-fold. The affinity of rsTF for FVIIa in the presence of C6PE (*K*_d_ = 1.3 ± 0.5 nM) was observed to be similar to that in the presence of C6PS (*K*_d_ = 2.6 ± 0.6 nM) [25]. Next, we collected data with FVIIa and sTF in the presence of a fixed concentration of C6PS followed by the titration with increasing concentrations of C6PE and *vice versa*. Our result (data not shown here) indicate cooperativity of FVIIa activation of FX in the presence of rsTF similarly like in the absence of rsTF.

A reasonable interpretation of this observation is that these two amino phospholipids regulate the cofactor activity of TF by promoting its tight binding to FVIIa. Since we observed that the Gla domain of FVIIa is important in determining both its activity and structural response to both C6PS and C6PE (Figures 1 and 2), we also examined the effect of the Gla domain on binding to rsTF, as recorded in Figure 3B. The absence of the FVIIa Gla domain reduced the affinity of rsTF for FVIIa only by roughly two-fold but reduced the increase in proteolytic activity by 70–80-fold. Thus, the FVIIa Gla domain is needed to form an optimal enzyme–cofactor–substrate complex.

### Effect of rsTF on the amidolytic activity of FVIIa and des Gla-FVIIa in presence lipid

**Figure 4:**
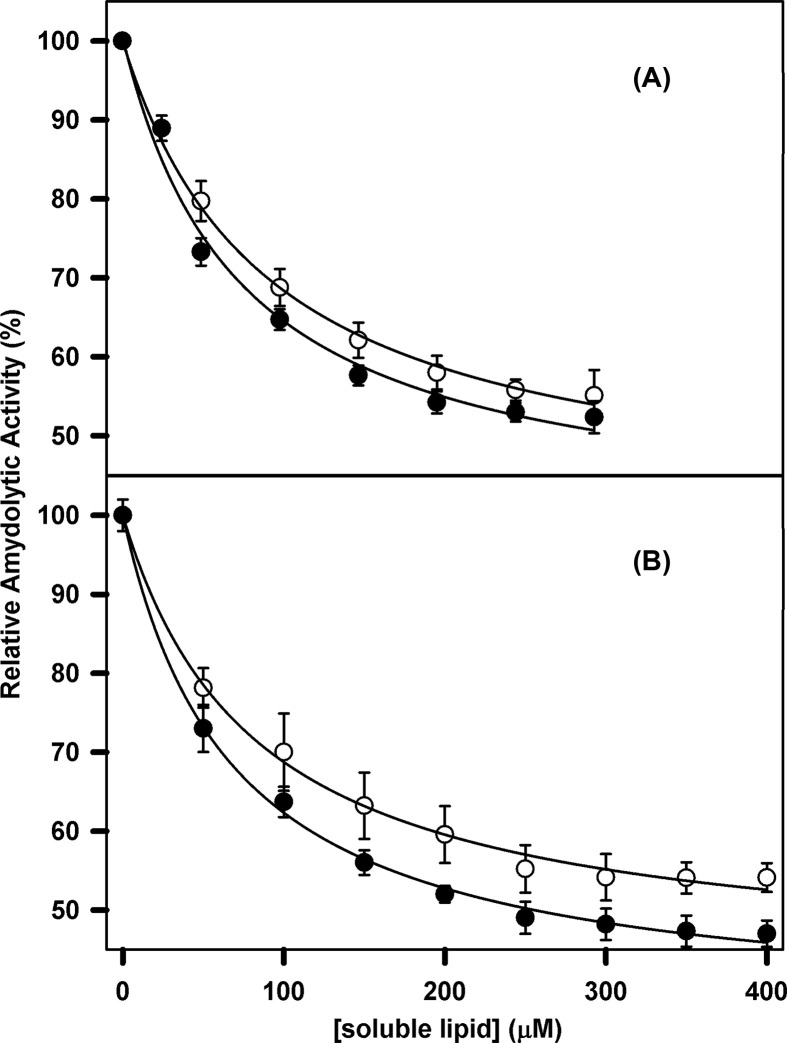
Effect of C6PS and C6PE on FVIIa amidolytic activity with or without rsTF (**A**) The amidolytic activity of 100 nM FVIIa in a buffer containing 20 mM Tris, 150 mM NaCl, 5 mM Ca^2+^ and 0.6% PEG was assayed in the presence of 1 mM substrate Leu-PHG-Arg-pNA.AcOH and increasing concentrations of C6PS (•) and C6PE (○). Fitting the data to a single-site binding model yielded a *K*_d_ of 72 ± 17 with C6PS and 93 ± 14 µM with C6PE. (**B**) The amidolytic activity of 25 nM FVIIa in the presence of 100 nM rsTF in a buffer containing 50 mM Tris, 150 mM NaCl, 5 mM Ca^2+^ and 0.6% PEG was assayed in the presence of 1 mM substrate Leu-PHG-Arg-pNA.AcOH and increasing concentrations of C6PS (•) and C6PE (○). Fitting the data to the binding model explained in Figure 1 yielded a *K*_d_ of 68 ± 5 μM with C6PS and 83 ± 9 µM with C6PE.

Proteolytic activity depends on binding of substrate (in this case FX) to both the active site and possibly to substrate-binding exosites [26]. In order to discern whether the lipid-induced increase in FVIIa proteolytic activity is due to an influence of lipid on the FVIIa active site or to binding of the substrate (FX) to an exosite, we titrated FVIIa amidolytic activity towards the synthetic substrate Leu-PHG-Arg-pNA.AcOH with both C6PS and C6PE. The results are shown in Figure 4. Analysis of the curves in Figure 4A is described in the Appendix according to a single-site model, with resulting parameters gathered in Table A5. Comparing Tables A4 and A5 makes it clear that FVIIa amidolytic and proteolytic activities respond with similar apparent *K*_d_’s to both C6PS and C6PE binding, although the relative amidolytic response is negative (decreases upon binding soluble lipids) while the proteolytic response is positive. rsTF had essentially no effect on the amidolytic response to soluble lipid binding while the proteolytic activity was increased 5-fold and 20-fold by C6PS and C6PE binding respectively. This suggests that association of FVIIa with rsTF enhances activity by promoting formation of a substrate-binding exosite rather than solely through enhancing active-site activity.

### Determination of stoichiometry of binding using a global analysis of all datasets

Our data show that PS and PE cause structural changes to FVIIa with apparent dissociation constant *K*_d_ ∼ 150–300 μM (Figure 1) and affect proteolytic and amidolytic activity with apparent *K*_d_ ∼ 50–100 μM (Figures 2 and 4). Since it is not possible to determine the number of PS/PE binding sites from single datasets, we analyzed all datasets simultaneously with a global fit, where each parameter in a model, such as *K*_d_, has to be equal for all datasets simultaneously. The details of the analysis are described below.

**Figure 5:**
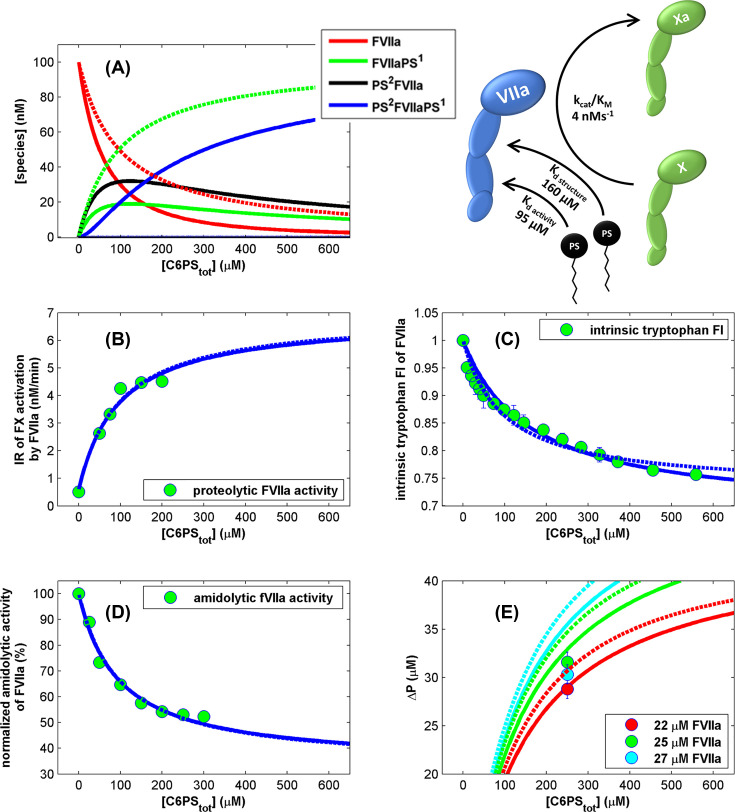
Regulation of FVIIa activity by soluble PS deploying global fitting and utilizing same parameters used in a two-independent-site model with four fitting parameters The best fit (Fit #3, $\bar{\chi^{2}}=1.57$) with n_1_ = 1, n_2_ = 1 is shown with solid lines, next best fit with n_1_ = 2, n_2_ = 0 (Fit #4, $\bar{\chi^{2}}=2.1$) is shown with dashed lines, fit with variable n_1_ and n_2_ (Fit #9, $\bar{\chi^{2}}=1.62$) is not shown, since it overlaps with the best fit #3: (**A**) concentrations of the protein species: free FVIIa (red), FVIIa with a C6PS bound to the first site (green), FVIIa with a C6PS bound to the second site (black), FVIIa with both C6PS sites occupied (blue); (**B**) FVIIa proteolytic activity; (**C**) FVIIa intrinsic tryptophan fluorescence intensity; (**D**) FVIIa amidolytic activity; (**E**) concentration of bound lipid (ΔP) as determined by equilibrium dialysis.

**Figure 6:**
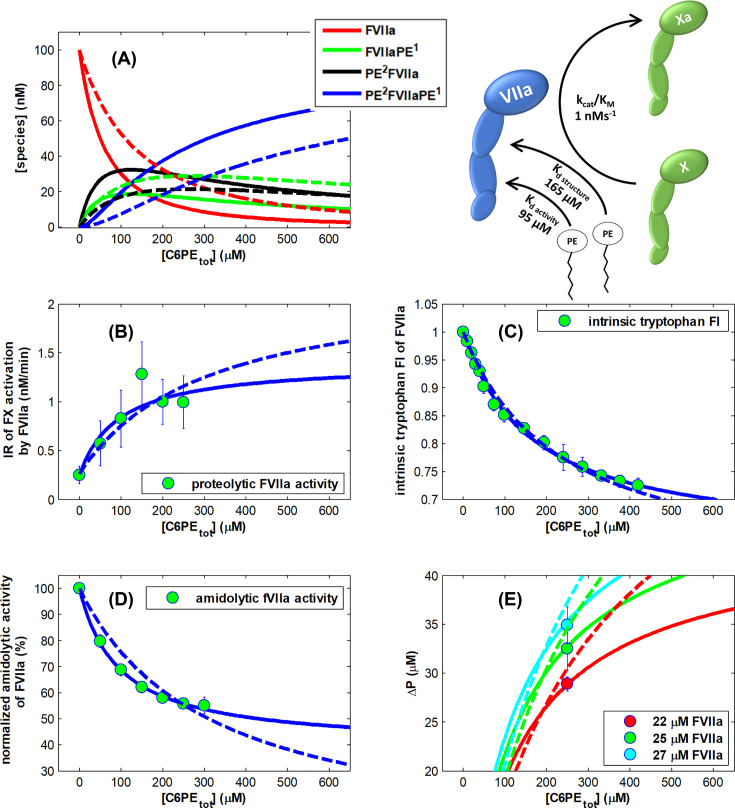
Regulation of FVIIa activity by soluble PE deploying global fitting and utilizing same parameters used in a two-independent-site model with four fitting parameters The best fit (Fit #3, $\bar{\chi^{2}}=0.47$) with n_1_ = 1, n_2_ = 1 is shown with solid lines, next best fit with n_1_ = 1, n_2_ = 2 (Fit #6, $\bar{\chi^{2}}=1.73$) is shown with dashed lines, fit with variable n_1_ and n_2_ (Fit #9, $\bar{\chi^{2}}=0.49$) is not shown, since it overlaps with the best fit #3: (**A**) concentrations of the protein species: free FVIIa (red), FVIIa with a C6PE bound to the first site (green), FVIIa with a C6PE bound to the second site (black), FVIIa with both C6PE sites occupied (blue); (**B**) FVIIa proteolytic activity; (**C**) FVIIa intrinsic tryptophan fluorescence Intensity; (**D**) FVIIa amidolytic activity; (**E**) concentration of bound lipid (ΔP) as determined by equilibrium dialysis.

We used the two-independent-site model, where we assumed that (n_1_ + n_2_) C6PS or C6PE molecules bind to two classes of FVIIa sites, as we did previously for C_6_PS binding to FIXa [12]. The model is briefly summarized here. For each class of sites, the site dissociation constants are assumed to be identical within that class (*K*_d,1_ and *K*_d,2_) and the sites are assumed to be independent. The data in Figures 1, 2C and 3 support this model, although these data cannot establish both the site-dissociation constants and stoichiometry of these two classes of sites when fit individually. However, a simultaneous fit of these three independent experimental datasets along with equilibrium dialysis data (Figure 5E and 6E) can establish the dissociation constants and stoichiometries. The stoichiometries of C6PS or C6PE binding to FVIIa must be natural numbers; therefore, we treated them as such in order to obtain the best fit. In addition, we also treated the stoichiometries as variable parameters, to test the appropriateness of the model.

Four processes define occupation of these sites:

1. binding of n_1_ PS molecules to site 1 of FVIIa to produce the species $FVIIaPS_{n_{1}}^{1}$,
2. binding of n_2_ PS molecules to site 2 of FVIIa to produce the species $PS_{n2}^{2}FVIIa$,
3. binding of n_2_ PS molecules to $FVIIaPS_{n_{1}}^{1}$ to produce the species $PS_{n_{2}}^{2}FVIIaPS_{n_{1}}^{1}$, and
4. binding of n_1_ PS molecules to $PS_{n2}^{2}FVIIa$ to produce the species $PS_{n_{2}}^{2}FVIIaPS_{n_{1}}^{1}$.

Subscripts indicate the number of ligands binding to the class of sites indicated by the superscript. Assuming that on- and off-rates are sufficiently fast to establish equilibrium and that ligands bind independently to sites 1 and 2 (i.e., no linkage between sites), the different FVIIa species that result from these four processes are defined by the following equilibria and binding equations:

(1) $$FVIIa+n_{1}PS⇄FVIIaPS_{n_{1}}^{1}$$

(2) $$FVIIa+n_{2}PS⇄PS_{n_{2}}^{2}FVIIa$$

$$K_{d,2}=\frac{\left[VIIa][PS\right]}{\left[PS_{n_{2}}^{2}VIIa\right]}$$

Because of site independence, there are only two distinct site-binding constants. In (eqns 1 and 2), *K*_d,1_ is the equilibrium dissociation constant for C6PS binding to the first class of equivalent and independent PS binding sites on FVIIa, and, in (eqns 3 and 4), *K*_d,2_ is the equilibrium dissociation constant for binding of C6PS binding to the second class of PS binding sites.

We applied the two-independent-site model to simultaneously fit four independent experimental datasets presented in (Figures 1, 2C, 3 and 5E) to determine stoichiometry of these two classes of sites and the dissociation constants.

The quantity ΔP measured by equilibrium dialysis (shown in Figures 5 and 6) is the difference in ligand concentration between two compartments and was fitted using the equation described in more detail previously [12]:

(3) $$\Delta P=[FVIIa]\cdot \frac{[PS]\cdot K_{1}\cdot n_{1}+[PS]\cdot K_{2}\cdot n_{2}+(K_{1}\cdot n_{1}+K_{2}\cdot n_{2})\cdot {\left[PS\right]}^{2}}{1+[PS]\cdot K_{1}+[PS]\cdot K_{2}+{\left[PS\right]}^{2}\cdot (K_{1}+K_{2})},$$

where [FVIIa], and [PS] are concentration of free FVIIa and C6PS species as predicted by the model. *K*_i_’s are association constants (1/*K*_d,i_) for each of the two classes of binding sites.

The intrinsic tryptophan fluorescence intensity (*F*) in the presence of soluble PS can be written as:

(4) $$F=[FVIIa_{free}]\cdot F_{FVIIa_{free}}+[FVIIaPS^{1}]\cdot F_{FVIIaPS^{1}}+[PS^{2}FVIIa]\cdot F_{PS^{2}FVIIa}+[PS^{2}FVIIaPS^{1}]\cdot F_{PS^{2}FVIIaPS^{1}},$$

where [*FVIIaPS*^1^], [*PS*^2^*FVIIa*], [*PS*^2^*FVIIaPS*^1^] are concentrations of FVIIa species as predicted by the model, and *F_FVIIa free_* represents the molar intensity of FVIIa fluorescence in solution, $F_{FVIIaPS^{1}}$ and $F_{PS^{2}FVIIa}$ are the molar intensities associated with FVIIa having C6PS bound to either the first or second class of C6PS binding sites, respectively. Note that we make the simplifying assumption that the fluorescence intensity is independent of how ligands (i.e., *n_i_*) bind to each class of sites. Similarly, $F_{PS^{2}FVIIaPS^{1}}$ is the fluorescence intensity of FVIIa with C6PS bound to both classes of sites. The fluorescence intensity signal from a FVIIa species (*F_i_*) depends on both the concentration ([FVIIa]_i_) and the molar intrinsic intensity of each species *i*.

In order to describe proteolytic activity of FVIIa against FX in the presence of increasing concentration of C6PS (shown in Figure 2), we used the classical form of the Michaelis–Menten equation:

(5) $$\frac{dP}{dt}=k_{cat}[FVIIa_{total}]\frac{\left[FX\right]}{K_{M}+[FX]}.$$

Since *K*_m_ of FX activation by FVIIa is 11.4 μM [27], and the substrate [FX*_total_*] = 0.3 μM, these conditions satisfy [FX*_total_*] << *K*_M_ and the Michaelis–Menten equation for the initial rate of FX activation transforms into:

(6) $$\frac{dP}{dt}=\frac{k_{cat}}{K_{M}}[FVIIa_{total}][FX_{total}],$$

where [FX_total_] = 300 nM, and [E_total_] = [FVIIa _total_] = 100 nM in our experiments.

Since different FVIIa species can have different activities, we can write observed activity as:

(7) $$R=\frac{dP}{dt}=\sum_{i}{\left(\frac{k_{cat}}{K_{M}}\right)}_{i}[{\text{FVIIa}}_{i}][{\text{FX}}_{total}],$$

where the index *i* represents one of four forms of the enzyme. Then, as for structural properties described above, the observed initial rate is the sum of four rates, where *k*_cat,i_ and *K*_M_ are the usual kinetic constants in this formalism, and [FVIIa] and [FX_total_] are the concentrations of FVIIa and FXa, respectively.

We solved here a set of equations numerically using MATLAB, version R2011b for any given set of parameters (n_1_, n_2_, *K*_1_, and *K*_2_). Using the equations presented above, we predicted: (a) concentrations of each FVIIa species, (b) initial rates of FX activation by FVIIa, (c) amydolitic activity of FVIIa, (d) intrinsic tryptophan fluorescence of FVIIa, and (e) ΔP as a function of total soluble lipid concentration. This requires 16 additional unknown intensive parameters (molar quantities in eqns 3–7), but these can be fixed by observation as previously in the case of FIXa [12]. First, we note that only the weak class of sites affects structural properties, so F1 and F2 (= F4) are fixed as the inital and asymptotic observables for both intrinsic fluorescence and, while F3 = F1 (i.e., occupancy of the tighter class of sites does not influence intrinsic fluorescence, Figure 1). Second, only occupancy of the tighter class of sites affects activity, so *k*_cat,1_ and *k*_cat,3_ (=*k*_cat,4_) are establihed as the inital and asymptotic values from Figure 3 for both proteolytic and amidolytic activity, while *k*_cat,2_ = *k*_cat,1_, i.e., the activity of free FVIIa was set to be negligible, i.e. 0. There are no extensive parameters associated with the observable ΔP. These predicted observables were compared with the experimental data to calculate the sum of the squares of deviations of calculated values from experimental data (χ^2^). We minimized this with respect to the unknown dissociation constants and stoichiometries to obtain estimates of these parameters that allow us to describe all four datasets simultaneously (i.e., globally). Dividing the minimized χ^2^ by the number of degrees of freedom (#data points - #parameters -1) yields the reduced chi-squared ($\bar{\chi^{2}}$), which should be close to 1 for an adequate description of the data by the model (if number of data points is much larger than the number of fitting parameters). For simplicity, all observables were expressed as ratios of values in the presence of C6PS to those for free FVIIa, i.e., the fluorescence recorded at the beginning of each experiment. Best fits with different assumed stoichiometries n_1_ and n_2_ are summarized in Table 1 (for C6PS) and Table 2 (for C6PE).

**Table 1:** Results of globally fitting four datasets (FVIIa proteolytic and amidolytic activity, FVIIa intrinsic tryptophan fluorescence intensity, from Figures 1-3, and equilibrium dialysis data versus concentration of total C6PS) to the two-independent-site model with four global fitting parameters (best fit in bold)

Fit #	n_1_	n_2_	n_1_+n_2_	$\bar{\chi^{2}}$	K_d,1_ (μM)	K_d,2_ (μM)	Proteolytic activity of *PS*^2^*FVIIa k*_cat_/*K*_M,3_ (10^9^ M^−1^s^−1^)	Relative amidolytic activity of *PS*^2^*FVIIa* (%)	Relative intrinsic fluorescence intensity of *PS*^1^*FVIIa* (%)
1	1	0	1	3.7	46	3	46	77	
2	0	1	1						
3	1	1	2	**1.57**	**159**	**94**	**3.8**	**34**	**69**
4	2	0	2	2.1	97	3.8	33	73	
5	0	2	2						
6	1	2	3	2.9	252	242	6	0	62
7	2	1	3	2.4	440	99	3.9	32	51
8	2	2	4	3.7	840	220	5.7	5	27
									
9	1.0	0.94	1.94	1.62	148	91	3.8	34	69

Relative amidolytic activity and relative intrinsic fluorescence intensity are shown as percent of the activity and the fluorescence intensity of free FVIIa. The *k*_cat_/*K*_M_ of FVIIa without C6PS bound was estimated by the best fit to 0.32 nM/s.

The best fit with $\bar{\chi^{2}}=1.57$ was obtained for stoichiometry of n_1_ = 1 and n_2_ = 1 is shown in Figure 5.

**Table 2:** Results of globally fitting four datasets (FVIIa proteolytic and amidolytic activity, FVIIa intrinsic tryptophan fluorescence intensity, from Figures 1-3, and equilibrium dialysis data *versus* concentration of total C6PE) to the two-independent-site model with four global fitting parameters (best fit in bold)

Fit #	n_1_	n_2_	n_1_+n_2_	$\bar{\chi^{2}}$	*K*_d,1_ (μM)	*K*_d,2_ (μM)	Proteolytic activity of *PE*^2^*FVIIa k*_cat_/*K*_M,3_ (10^9^ M^−1^s^−1^)	Relative amidolytic activity of *PE*^2^*FVIIa* (%)	Relative intrinsic fluorescence intensity of *PE*^1^*FVIIa* (%)
1	1	0	1	5.2	38	0.63	51	76	
2	0	1	1						
3	1	1	2	**0.47**	**164**	**95**	**0.87**	**39**	**62**
4	2	0	2	2.01	116	0.83	35	67	
5	0	2	2						
6	1	2	3	1.73	230	310	1.28	0	56
7	2	1	3	1.75	375	173	0.97	23	43
8	2	2	4	2.87	705	306	1.27	0	16
									
9	1.12	0.92	2	0.49	165	97	0.78	39	62

Relative amidolytic activity and relative intrinsic fluorescence intensity are shown as percent of the activity and the fluorescence intensity of free FVIIa. The *k*_cat_/*K*_M_ of FVIIa without C6PE bound was estimated by the best fit to 0.14 nM/s.

The best fit with $\bar{\chi^{2}}=0.47$ was obtained for stoichiometry of n_1_ = 1 and n_2_ = 1 and is shown in Figure 6.

Majumder et al. have previously shown that two molecules of PS bind at two different sites on FIXa with different affinities (∼100-fold) that they appear to be independent sites [12]. A high affinity binding site (*K*_d_ ∼ 1.4 µM) regulates structure, whereas a low-affinity binding site (*K*_d_ ∼ 140 µM) regulates activity. Similarly, the activity-regulating PS binding site in FXa has dissociation constant *K*_d_ ∼ 80 μM while the other site that regulates structure has a *K*_d_ (200–600) µM [16,19].

The best global fit to all datasets, including equilibrium dialysis measurements (Figure 5 for PE, and Figure 6 for PS), indicates that FVIIa is regulated, just like FIXa, by independent binding of two molecules of PS. One binding site regulates activity *(K*_d_ ∼ 90 μM), while the other one regulates structure (*K*_d_ ∼ 160 μM). Different parameters of structure and activity regulation of factors VIIa, IXa and Xa by the soluble lipids are documented in Table 3.

**Table 3:** Comparison of soluble lipid regulation of FVIIa, FIXa, and FXa showing that the activity of all the coagulation factors considered is regulated by soluble lipid binding to a site with *K*_d_ ∼ 100 µM, while binding to the structure regulating site differs, as does the effect on the activity increase

	Activity regulating site *K*_d_ (μM)	Activity increase in presence of soluble lipids	Activity increase in presence of sTF	Structure regulating site *K*_d_ (μM)
FXa	70-90	200-fold	/	200–600
FIXa	142	13-fold	/	1.4
FVIIa	90	12-fold	100-fold	160

In summary, there are apparent differences in structural response of FVIIa, FIXa, and FXa by soluble short-chain lipids, whereas binding of the lipids to activity regulating site seems to be preserved (Table 3). Understanding of soluble lipid (lysolipid) regulation of serine proteases might be of great physiological significance, since lysophosphatidylserine (lyso-PS) is involved in coagulation and in early stages of initiating acute inflammation as well as in its resolution [28].

## Discussion

The present study, which is mainly focused on exploring the details of the soluble lipid binding to FVIIa, revealed new and unexplored facts. We studied the interaction of soluble lipids (mainly PS and PE) with FVIIa and FVIIa–rsTF without the presence of any membrane surface. Mostly, previous studies described the interaction of FVIIa with phospholipid membrane in the presence of its cofactor TF. However, our study is the first to describe the interaction of soluble phospholipid with FVIIa alone and to decipher the particular roles of phospholipids in regulating FVIIa.

In addition, our study will be useful because C6PS and C6PE are a perfect tool to study the effects of lysolipids. Lysolipids are an emerging class of signaling lipids involved in inflammatory and autoimmune diseases [28]. Activated platelets secrete Lyso-PS [29] and lyso-PE [30] *in vitro*. Lyso-PS may be involved specifically in platelet activation *in vivo*, [31] yet its physiological function has not been fully elucidated. Lysolipids occur in relatively high concentrations in blood plasma. However, it is difficult to investigate lysolipid activities in vitro because they tend to aggregate due to their sub-micromolar CMCs. Conversely, short-chain soluble lipids such as C6PS and C6PE have much higher CMCs, thus, they can be used to probe the lysoPS-binding site in the Gla domain. This site binds the lyso-PS headgroup and only the first four to six carbon atoms of the lyso-PS alkyl chain. Binding involves multiple types of interactions, e.g., calcium coordination, ionic bonds, and van der Waals and hydrophobic contacts [29].

The intrinsic tryptophan fluorescence of FVIIa in the presence of both C6PS and C6PE resulted in a simple hyperbola and we used a single-site binding model (Eqn 3 in Appendix) to fit the data. However, we could not detect any noticeable change in the overall secondary structure of FVIIa in the presence of soluble lipids from the CD spectroscopic measurement (data not shown). Notably, Taboureau et al. also reported no perturbation in FVIIa due to the insertion of C6Lyso-PS [30]. Both single and two lipid titrations with des Gla-FVIIa revealed the fact that at least one of the two lipid binding sites exists in the Gla domain.

Apparently, FVIIa has two different binding sites for PS and PE that regulate the activity of FVIIa. However, both PS and PE bind with similar affinity to their respective sites. When both sites are occupied, the activity of FVIIa is greater than with only one site occupied. Thus, PE in the presence of PS was most effective in enhancing the proteolytic effect. Increased FX activation in presence of PE containing membrane with little amount of PS (as low as 3 mol%) was also reported by Neuenschwander et al.; although PE dependency slowly diminished as PS concentration was increased [31].

Our data confirmed that the *K*_d_’s of binding to these activity-regulating sites are different from those determined by tryptophan fluorescence experiments. This indicates that the site regulating the activity of FVIIa and the site that regulates FVIIa structure are not same. Effect of PS and PE on FVIIa proteolytic activity was shown to be enhanced appreciably in presence of rsTF (Figure 2C). This might be due to stronger interaction of the enzyme (FVIIa) and cofactor (TF) in presence of the phospholipids as was observed for other coagulation factors too [10,11,20,32,33]. Removal of Gla domain, however, made FVIIa inactive with the affinity of rsTF towards des Gla–FVIIa (*K*_d_ = 2 nM) remained almost same. This clearly indicates that both PS and PE promote strong interaction between rsTF and des Gla-FVIIa by binding to rsTF rather than to FVIIa. Therefore, most of the free energy of interaction of FVIIa and rsTF is independent of the presence of the Gla domain. Lawson et al. also demonstrated that the increase in catalytic efficiency of FVIIa when complexed with TF was independent of the supporting surface [34]. A previous study showed that the rsTF–FVIIa complex did not bind to surface immobilized, mixed phosphatidylcholine-PS [17]. We made a similar observation with the soluble phospholipid.

Therefore, although the Gla domain of FVIIa is absolutely required for binding of soluble phospholipid to activate FVIIa, that domain is not necessary for binding of rsTF to FVIIa. When we determined the amidolytic activity of FVIIa in the presence of increasing concentration of PS and PE, we obtained site dissociation constants similar to those obtained from proteolytic activity measurements (Figure 4). We also observed PS to be more effective in promoting the activity of FVIIa compared with PE. When we performed the proteolytic activity assay in the presence of rsTF, we did not observe any change in apparent *K*_d_’s (68 ± 5 μM with C6PS and 83 ± 9 µM with C6PE). This suggests that association of FVIIa with rsTF enhances proteolytic activity mostly by promoting the binding of the substrate (FXa) to FVIIa exosite, and not by regulating the active site activity of FVIIa. This was supported further by the molecular dynamics (MD) simulation studies of the membrane-bound model of FVIIa–sTF complex [34]. MD simulation studies on membrane bound TF–FVIIa revealed that sTF interacts directly with the lipid headgroups and stabilizes the large-scale structural fluctuations of FVIIa due to the presence of the hinge regions between its domains. In the process, it also positions the catalytic triad of FVIIa in a more upright position with respect to the membrane than in isolated FVIIa. This spatial stabilization of the catalytic region of FVIIa by sTF ensures optimal interaction of FVIIa with its substrate FX. Reorientation of a clotting factor with respect to the membrane, induced by a cofactor, was also reported for the FXa-FVa complex [15]. In that case, C6PS bound both FXa and FVa to form an active complex. However, unlike FVIIa, FXa has three plausible C6PS binding sites [35] Apart from the one present in the Gla domain, the other two lipid binding sites are present in the epidermal growth factor domain and the catalytic domain. Binding of lipids to these three widely separated structural domains of FXa involves linkage between the sites. Similar regulation by C6PS was also observed for another serine protease of the coagulation cascade, FIXa. FIXa has two PS binding sites, each having a stoichiometry of 1 [12]. However, whether FVIIIa, the cofactor of FIXa, binds to lipid and brings in structural changes in FIXa is yet to be explored.

In summary, we have successfully used soluble lipids as an alternative for a phospholipid membrane to establish the importance of PS and PE in blood coagulation. The present study revealed that molecular PS and PE regulate FVIIa activity, but, unlike the Xa–Va complex, neither PS nor PE influence FVIIa–rsTF complex formation. Our conclusions are enumerated below:

1. PS and PE bind to one or more sites on FVIIa with equal affinities.
2. At least one of the lipid binding sites exists in Gla doman.
3. PS and PE act cooperatively to influence the proteolytic activity of FVIIa.
4. There is huge reduction in proteolytic activity of FVIIa without its Gla domain.
5. Soluble lipids trigger a strong association of rsTF, to FVIIa,
6. Increased proteolytic activity of FVIIa in presence of rsTF can be attributed to cofactor (TF) induced conformational change in FVIIa to facilitate its binding to its substrate, FXa.

**Figure 7:**
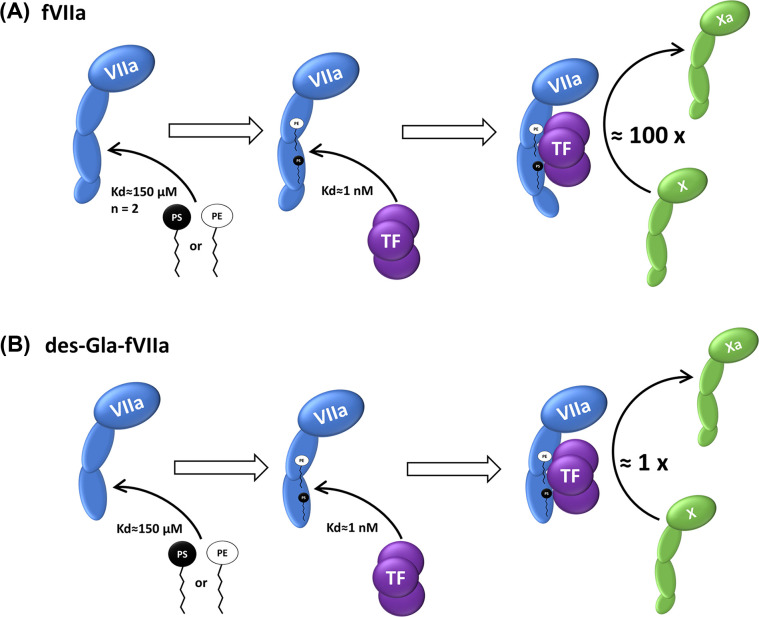
Soluble lipid regulation of FVIIa Schematic presentation summarizing the effect of soluble lipids on (**A**) FVIIa activity, and (**B**) des Gla-FVIIa. Although rsTF binds equally tightly (*K*_d_ = 2 nM) to FVIIa in presence of high amount of soluble lipids, Gla domain is needed for FVIIa to achieve physiologically relevant saturating activity (as shown in Figure 3A).

Finally, the essential findings of our study are summarized in Figure 7.

## Data Availability

All supporting data are included in the main text and supplementary files.

## Abbreviations

CMC: critical micelle concentration
C6PS: 1,2-dicaproyl-sn-glycero-3-phospho-l-serine
C6PE: 1,2-dicaproyl-sn-glycero-3-phospho-ethanolamine
des Gla-FVIIa: Gla-domainless factor VIIa
FVIIa: factor VIIa
FIX: factor IX
FX: factor X
Gla: γ-carboxyglutamic acid-rich domain
lyso-PS: lysophosphatidylserine
MD: molecular dynamics
PE: phosphatidylethanolamine
PS: phosphatidylserine
rsTF: recombinant soluble tissue factor
sTF: soluble tissue factor
S-2765: N-2-benzyloxycarbonyl-d-arginyl-l-arginine p-nitroanilide dihydrochloride
TF: tissue factor
